# Exploring possible benefits of *Litsea cubeba* Pers. extract on growth, meat quality, and gut flora in white-feather broilers

**DOI:** 10.3389/fvets.2023.1335208

**Published:** 2024-01-11

**Authors:** Yankai Luo, Yuchen Bi, Ziyun Xu, Linxian Shan, Jun He, Kedan Wang, Zhengjiang Zhou, Lihui Yu, Xingjiao Jiang, Jiangrui Yang, Lijun Yu, Rui Gao, Jingran Wei, Xiaocui Du, Yan Liu, Chongye Fang

**Affiliations:** ^1^College of Agronomy and Biotechnology, Yunnan Agricultural University, Kunming, China; ^2^Yunnan Research Center for Advanced Tea Processing, Yunnan Agricultural University, Kunming, China; ^3^College of Food Science and Technology, Yunnan Agricultural University, Kunming, China; ^4^College of Tea, Yunnan Agricultural University, Kunming, China; ^5^The International College, Yunnan Agricultural University, Kunming, China

**Keywords:** *Litsea cubeba* (Lour.) Pers., meat quality, gut microflora, white-feather broiler, natural feed additive

## Abstract

White-feather broiler chickens are the dominant species in global poultry meat production. Yet there is growing concern about their health, quality, and growth efficiency. While feed additives, often antibiotics or synthetic chemicals, are used to maintain the health of the animals, drug resistance limits their use. *Litsea cubeba* (Lour.) Pers., a traditional Chinese herb with antibiotic-like benefits but without the risk of drug resistance, has not yet been explored as an additive to broiler diets. In the present study, broilers of the AA+ hybrid strain were randomly divided into three groups of 16: a control group (regular feed), a low-dose group (1.25 g/kg added *L. cubeba* extract), and a high-dose group (2.50 g/kg added *L. cubeba* extract). After 35 days, we found that the extract had no effect on growth. However, gut flora analysis revealed that both doses of the extract had a positive influence on amino acid content and minor unsaturated fatty acids, thus improving the flavor and nutritional value of the meat. These findings suggest that *L. cubeba* extract, at either dose, could serve as a sustainable alternative to antibiotics, thus reducing the risk of drug resistance while improving meat quality, nutrition, and flavor.

## 1 Introduction

White-feather broiler chickens have become the dominant species in global poultry meat production because of their fast growth rates, high feed-to-meat conversion ratios, and short growth times (1). Globally, China ranks second in poultry production and consumption (2, 3); therefore, with an increasing public focus on better living standards and health awareness, meat quality has become a key concern for producers and consumers (4). Because most characteristics of meat quality are influenced by various genes, environmental factors, and their interactions, improving meat quality through short-term genetic breeding is a difficult task (5). However, the use of specific feed additives can considerably improve meat quality in a short period.

Enhancing broiler chicken growth and development can be achieved through judicious use of antibiotics (6). However, the extended withdrawal period, which can exceed 10 days for commonly used quinolone antibiotics, frequently results in the persistence of antibiotic residues, the emergence of antibiotic resistance, disturbances in the gut microbiota of processed broilers, and environmental antibiotic pollution (7–9). Therefore, to improve livestock and poultry feed sustainably and naturally, it may be advantageous to use medicinal plants containing active phytochemicals with no antibiotic residues or pollutants (10). Chinese herbal medicine has been used for centuries to prevent and treat illnesses and enhance animal growth while avoiding the potential antibiotic resistance risk resulting from drug overuse (11, 12). For example, an investigation into the effects of *Astragalus membranaceus* root powder on growth performance, carcass characteristics, and antioxidant enzymes and metabolites in the blood and liver of white-feather broilers showed that adding 10,000 mg/kg of root powder to the feed significantly improved daily weight gain and feed conversion efficiency (13). Similarly, Liu et al. (14) demonstrated that the addition of 1,000 mg/kg of betaine to feed effectively alleviated the adverse effects of heat and transportation stress on weight gain, feed intake, and muscle quality of white-feather broilers. Therefore, herbal additives are promising green alternatives to antibiotics and synthetic chemicals in foods used to maintain the health status of poultry, preserve the normal functioning of the gastrointestinal tract, optimize poultry production performance, improve meat quality and flavor, and improve economic benefits (15, 16). Consequently, in-depth explorations of the potential applications of herbal products and their extracts as feed additives can contribute to the sustainable development of the broiler industry.

*Litsea cubeba* (Lour.) Pers. is an evergreen tree or shrub native to southern China and Southeast Asia with a long history of use in traditional Chinese medicine (17, 18). The fruits or extract of *L. cubeba* have multiple applications in pharmaceutics, medicine, and food production, such as in spice formulations to enhance flavor and as a precursor for vitamin A synthesis (19, 20). The main bioactive phytochemicals in *L. cubeba* are secondary metabolites, including phenolic compounds, flavones, isoflavonoids, and flavonoids, with antioxidant, anti-inflammatory, and antitumor effects (21). The main pharmacological effects of the plant include relief from epigastric discomfort, reduced vomiting and hiccups, and enhanced immune function (19). For example, Goldar et al. (22) found that the fruit of *L. cubeba* significantly alleviated diabetic complications in mice. However, to the best of our knowledge, *L. cubeba* has not been tested as an additive to improve broiler health and meat quality. Therefore, the objective of this study was to investigate the effects of *L. cubeba* fruit extract on the growth performance of white-feather broiler chickens and the quality and flavor of their breast muscle. In addition, we sought to further elucidate the potential underlying mechanisms by analyzing associations with the cecum microbiota.

## 2 Materials and methods

### 2.1 Preparation of *Litsea cubeba* fruit extract

The fruits of *L. cubeba* were purchased from the “Yunnan Plateau Fruit and Vegetable Direct Delivery” online store in Taobao, China. Subsequently, the samples were subjected to professional authentication by Professor Chongye Fang of the College of Food Science and Technology, Yunnan Agricultural University.

**Figure 1:**
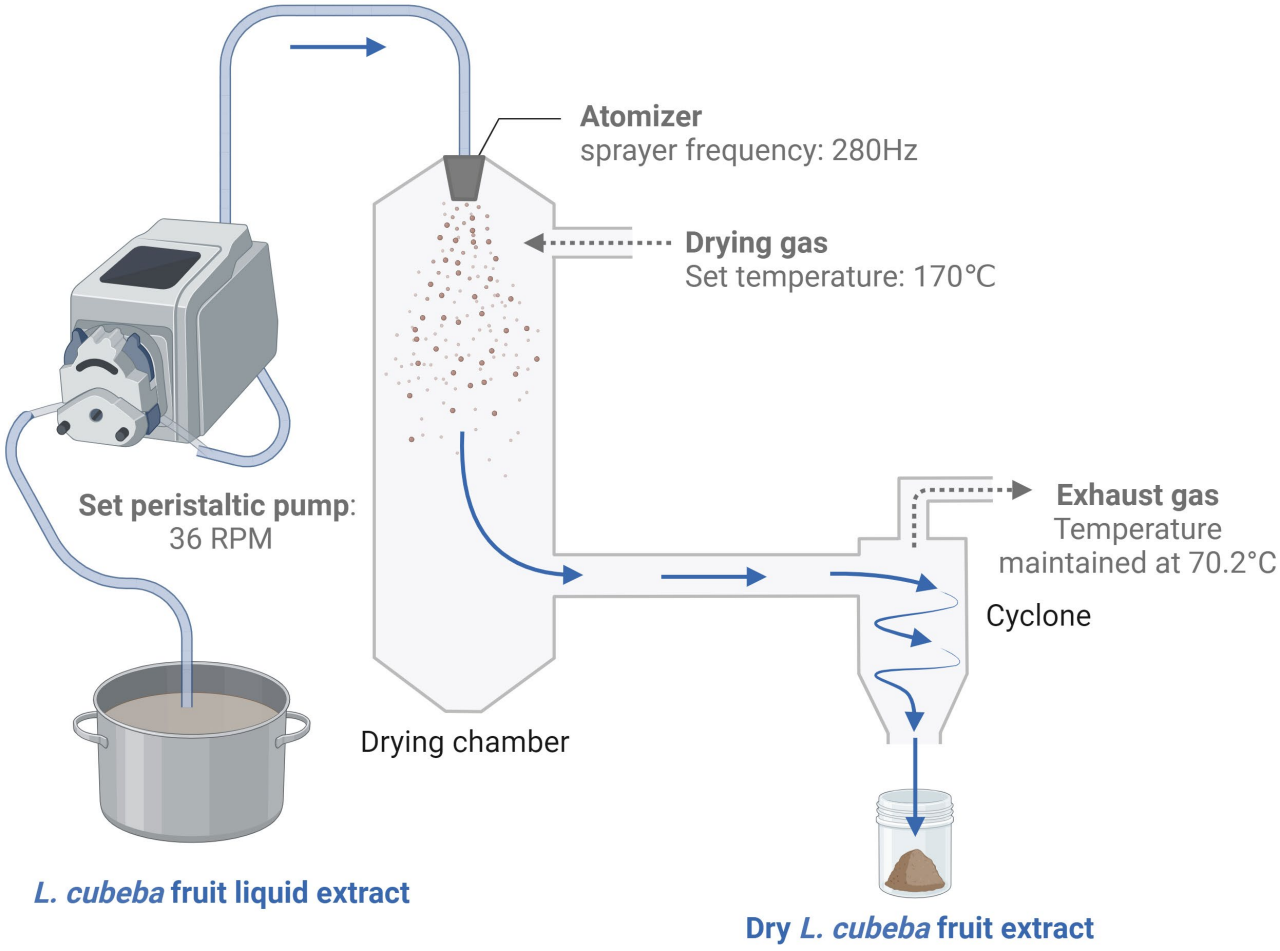
*Litsea cubeba* fruit extraction process. The *L. cubeba* fruit was coarsely crushed and extracted using purified water in a sample-to-solvent ratio of 1:10. The extraction was conducted on an electromagnetic stove at a constant temperature of 90°C. The solution was then filtered three times through steel wire mesh and nylon cloth, and the combined filtrates were concentrated to one-fifth of their original volume to obtain a condensed extract of *L. cubeba* fruit. Subsequently, this concentrated solution was subjected to spray drying, with a product recovery rate of 6.04%. The image was created on BioRender.com.

To prepare the extract, 1.5 kg of *L. cubeba* fruit was pulverized using an electric food processor (model YB-4500A, Yongkang Sufeng Trading Co., Ltd., China) until a granule size that could pass through a 60-mesh wire sieve was achieved. The samples were then extracted with purified water in a sample-to-solvent ratio of 1:10 for 30 min at 90°C. The resulting liquid was filtered through a 100-mesh wire sieve, followed by subsequent filtrations through 200-mesh and 600-mesh nylon screens to obtain the final liquid extract. The solid residue was then extracted twice using the same sample-to-solvent ratio as in the previous extraction. The pooled filtrate derived from three consecutive extractions was spray-dried (HF-5L spray dryer, Shanghai Hefan Co., Ltd.), with the air inlet temperature adjusted to 170°C, peristaltic pump calibrated to 36 rpm, sprayer frequency set at 280 Hz, and a resultant air outlet temperature of 70.2°C. This procedure yielded 90.56 g of powdered extract. This process was repeated three times to obtain the amount of powdered aqueous extract of *L. cubeba* fruit required for a comprehensive experiment. The *L. cubeba* fruit extraction process is schematically illustrated in Figure 1.

### 2.2 Experimental procedures involving animals

Forty-eight healthy, white-feathered broiler chickens of the AA+ hybrid strain, each with an average initial body weight of 37 g, were obtained at approximately 3 days of age from Hunan Shuncheng Industrial Co., Ltd. in China. These chickens were individually housed in two separate cages, ensuring controlled and consistent rearing conditions. The chickens were randomly divided into three groups of 16 each: a control group (CON), a low-dose group (L), and a high-dose group (H). All chickens were reared in stainless-steel cages equipped with feed and water troughs, maintaining a stocking density not exceeding 30 kg/m^2^. The nutritional content of the basal diet was formulated to vary with the different growth stages. For the early stage, crude protein and metabolizable energy were 21.04% and 12.33 MJ kg^−1^, respectively, while for the late stage, crude protein and metabolizable energy were 18.97% and 12.76 MJ kg^−1^, respectively. Considering the longer transportation time for the chicks, a 4-day adaptation period was provided to all the chickens, during which they consumed a base diet. The experiment was then started when the chickens reached 7 days of age. The control group received only the base diet and had free access to drinking water. The low- and high-dose groups received feed supplemented with 1.25 g/kg and 2.5 g/kg of *L. cubeba* fruit extract, respectively. The experiment lasted for 35 days and was divided into an early stage (days 7–21) and a late stage (days 22–42). The basic formulation of the feed followed the “Feeding Standards of Chicken” guidelines (NY/T33-2004, China) and met the NRC1994 amino acid requirements for broiler chickens. The specific composition and nutritional levels of the base diet are presented in Table 1.

**Table 1:** Composition and nutritional profile of base diet.

Ingredients	Early growth stage (days 7–21), %	Late growth stage (days 22–42), %
Corn	58.50	60.10
Limestone	1.60	1.35
Soybean oil	3.04	4.50
Soybean meal	28.00	30.00
Fermented soybean meal	5.00	—
Sodium chloride	0.22	0.26
Threonine	0.10	0.09
Lysine	—	0.10
Methionine	0.14	0.15
Calcium monohydrogen phosphate	1.40	1.45
Premix^a^	2.00	2.00
Total	100	100
**Nutrients**		
Metabolizable energy^b^/MJ kg^−1^	12.33	12.76
Crude protein	21.04	18.97
Crude fat	5.60	7.06
Calcium	1.00	0.90
Methionine	0.43	0.40
Lysine	1.08	1.01
Total phosphate	0.65	0.60

a The premix contained the following components per kg: retinol (9,500 IU), cholecalciferol (500 IU), α-tocopherol (20 IU), phylloquinone (1.2 mg), thiamine (2.2 mg), riboflavin (5.0 mg), pyridoxine (2.0 mg), nicotinamide (30 mg), calcium pantothenate (12.0 mg), folate (0.8 mg), d-biotin (0.18 mg), iodine (elemental, 0.35 mg), selenium (elemental, 0.30 mg), manganese (elemental, 100 mg), iron (elemental, 80 mg), copper (elemental, 8 mg), and zinc (elemental, 75 mg). The premix contained no antibiotics or chemically synthesized antibacterial agents. The values presented are measured quantities in percentages.

b The values for metabolizable energy were computed, while the levels of other nutrients were determined through measurement.

The experiment was conducted at an experimental chicken farm at the College of Animal Science and Technology, Yunnan Agricultural University. One week before the experiment, the chicken houses were disinfected and cleaned. The experimental chicken house was semi-enclosed, and the temperature, humidity, and ventilation were manually controlled. The initial temperature was set at 32°C and gradually decreased by 2–3°C per week until it reached 20°C. Throughout the experiment, the chicken houses were illuminated 24 h per day. The chickens were fed via plastic feeders and provided drinking water in plastic buckets once daily, and their daily feed and water intake were recorded. The body weights of the chickens were measured weekly. At 7 days of age, the chickens were vaccinated against Newcastle disease via eye drops, and at 12 days of age, they were vaccinated against infectious bursal disease via water administration. In addition, strict adherence to routine management procedures was ensured to maintain good hygiene in the chicken houses.

### 2.3 Growth performance indices and collection of meat samples

Growth performance indices and meat samples were processed according to the procedures described by Li et al. (23).

#### 2.3.1 Growth performance indicators

The plastic feeding trays and water buckets were weighed every morning. Broiler body weights (numbered 001–048) were measured every 7 days. Using these data, we calculated the average daily gain [ADG; Eq. (1)] for each group of chickens. We also recorded the daily feed intake and the amount of leftover feed to calculate the average daily feed intake [ADFI; Eq. (2)] and the feed-to-gain ratio [F/G; Eq. (3)] for each broiler group. All pertinent indices are expressed in grams or days, as appropriate.


(1) $$ADG=\frac{\left(\mathrm{Final}\;\mathrm{weight}-\mathrm{Initial}\;\mathrm{weight}\right)}{\mathrm{Number}\;\mathrm{of}\;\mathrm{experimental}\;\mathrm{days}}$$

(2) $$\mathrm{ADFI}=\frac{\mathrm{Total}\;\mathrm{feed}\;\mathrm{intake}}{\mathrm{Number}\;\mathrm{of}\;\mathrm{experimental}\;\mathrm{days}}$$

(3) $$F/G=\frac{\mathrm{ADFI}}{ADG}$$

#### 2.3.2 Collection of poultry meat samples from white-feather broilers

At 42 days of age, the white-feather broilers were weighed in the morning. The feed was withdrawn at 2 A.M., followed by a 12 h fasting period with water access. Five broilers were randomly selected from each group. The birds were euthanized through exsanguination using a conventional neck cut. The *pectoralis major* muscle, or breast muscle, was harvested. The breast muscles were later divided into left and right sections. The right section was immediately placed in a moisture-resistant, self-sealing preservation bag and stored in a 4°C refrigerator for subsequent analyses of crude protein and fat content. Concurrently, tissue samples (weighing approximately 2 g) from the same region of the left breast muscle were collected and stored in cryovials. These were flash frozen in liquid nitrogen for 15 min and then transferred to a −80°C freezer for later analysis of standard amino acids and fatty acids within the muscle tissue.

### 2.4 Meat quality evaluation

#### 2.4.1 Measurement of slaughter performance

On day 42 of the experiment, the broilers were fasted for 12 h before slaughter (with water provided *ad libitum*), and their pre-slaughter live weights were recorded. From each treatment group, five white-feather broilers were randomly selected, and their identification numbers were recorded before slaughter. The slaughter performance was evaluated using the dressing, semi-eviscerated, and eviscerated percentages according to the Chinese National Standard NY-T 823-2020 (“Terminology and Measurement Methods for Poultry Production Performance”). Specifically:

1. The dressing percentage represents the ratio of the post-slaughter weight of the broilers after the removal of blood, feathers, and corneal layers of the feet, toe shells, and beak shells to their pre-slaughter weight when they were alive.
2. The semi-eviscerated percentage represents the percentage of the broiler’s weight remaining after further removal of the trachea, esophagus, intestines, cysts, spleen, bile duct, pancreas, reproductive organs, stomach contents, and horny membrane relative to its weight prior to being slaughtered.
3. The eviscerated percentage represents the percentage of the broiler’s weight after further removal of the heart, liver, stomach, lungs, abdominal fat, head, and feet relative to its weight prior to slaughtering.

Eqs. (4)–(6) were used to calculate these ratios.


(4) $$\mathrm{Dressing}\;\mathrm{percentage}=\frac{\mathrm{Dressing}\;\mathrm{weight}\;\left(g\right)}{\mathrm{Live}\;\mathrm{weight}\;\mathrm{before}\;\mathrm{slaughter}\;\left(g\right)}\times 100\%$$

(5) $$\mathrm{Demi}­\mathrm{eviscerated percentage}=\frac{\begin{array}{l} \mathrm{Semi}­\mathrm{eviscerated}\;\\ \mathrm{weight}\;\left(g\right) \end{array}}{\begin{array}{l} \mathrm{Live weight before}\;\\ \mathrm{slaughter}\;\left(g\right) \end{array}}\times 100\%$$

(6) $$\mathrm{Eviscerated}\;\mathrm{percentage}=\frac{\mathrm{Eviscerated}\;\mathrm{weight}\;\left(g\right)}{\begin{array}{l} \mathrm{Live}\;\mathrm{weight}\;\mathrm{before}\;\\ \mathrm{slaughter}\;\left(g\right) \end{array}}\times 100\%$$

#### 2.4.2 Measurement of meat pH

The right breast muscles of the broiler chickens were dissected using a scalpel and their pH was measured using a portable pH meter (Waterproof Portable pH Meter with 0.01 pH Resolution; model: HI9124; Hanna Instruments, Inc., China) pre-calibrated with three pH standard solutions of pH 4.01, 6.86, and 7.01. The pH meter was inserted into three locations within the right breast muscles, and measurements were taken at 45 min and 24 h post-mortem. The data were recorded once stable readings were displayed, and the average of three readings was calculated for each time point.

#### 2.4.3 Quantitative assessment of color attributes in meat products

The right breast muscles were carefully dissected with a scalpel and placed on a flat, white plastic tray. A colorimeter (Chroma Meters Measuring Head, model CR-410 Head; Konica Minolta Sensing Americas Inc., Japan) was pre-calibrated using white and black backgrounds. Three measurements were taken along the midline of the pectoral muscle, from the thickest to the thinnest sections, to determine luminance (L^*^), redness (a^*^), and yellowness (b^*^). The mean values of three measurements were calculated.

#### 2.4.4 Measurement of meat tenderness and cooking loss in meat products

A scalpel was used to remove a 5 cm-long muscle sample with a cross-sectional area of 1 cm^2^ from the breast muscles. The pre-cooking weight of each sample (g) was recorded. The muscle sample was then placed in a sealed bag in a water bath and heated to 80°C for 15 min. After cooling to 25°C–30°C, the sample was removed from the bag, excess surface moisture was removed with absorbent paper, and the weight after cooking was recorded. The cooking loss of the breast muscles was calculated using Eq. (7).


(7) $$\mathrm{Cooking loss}\;\left(\%\right)=\frac{\left(\begin{array}{l} Pre­\mathrm{cooking weight}\;\left(g\right)\\ -\mathrm{Post}­\mathrm{cooking weight}\;\left(g\right) \end{array}\right)}{Pre­\mathrm{cooking weight}\;\left(g\right)}\times 100\%$$

The cooked muscle samples were then subjected to shear force measurements using a digital meat tenderness meter (C-LM3, Northeast Agricultural University, China). The average of three shear force measurements was calculated.

#### 2.4.5 Measurement of water retention capacity in meat products

The chicken breast was weighed after being refrigerated at 4°C for 6 h. A 3 cm × 2 cm × 1 cm section of the breast muscle was excised using a scalpel, after which the pre-storage weight of the sample was obtained. The meat sample was then suspended in the 4°C refrigerator using a paper clip and, wrapped in cling film to prevent direct contact. After 24 h, the samples were weighed to determine their post-storage weights. The drip loss over the 24 h period was calculated using Eq. (8):


(8) $$\mathrm{Drip}\;\mathrm{loss}\;\left(\%\right)=\frac{\begin{array}{l} Pre­\mathrm{storage}\;\mathrm{weight}\;\left(g\right)\\ -\mathrm{Post}­\mathrm{storage}\;\mathrm{weight}\;\left(g\right) \end{array}}{Pre­\mathrm{storage}\;\mathrm{weight}\;\left(g\right)}\times 100\%$$

#### 2.4.6 Determination of crude protein and crude fat contents in meat products

The chicken breast was dissected with a scalpel and subsequently submitted to the Yunnan Province Product Quality Supervision and Inspection Research Institute for crude protein analysis, where the Kjeldahl nitrogen determination method was used, as specified in the Chinese National Standard for Food Safety GB5009.5-2016 (“Determination of Protein in Food”). Crude fat was quantified using the Soxhlet extraction method following the Chinese National Standard for Food Safety GB5009.6-2016 (“Determination of Fat in Food”).

#### 2.4.7 Determination of ash content and moisture content in meat products

The ash content of the broiler meat was determined according to the Chinese National Standard for Food Safety GB 5009.4-2010 (“Determination of Ash Content in Foods”), specifically for legumes and their higher phosphorus content, meat and poultry, egg, aquatic, and dairy products. Moisture content was measured using the direct drying method described in the Chinese National Standard for Food Safety GB 5009.3-2010 (“Determination of Moisture in Foods”).

#### 2.4.8 Determination of amino acid and fatty acid content in meat products

After the white-feather broilers were euthanized, bled, plucked, and eviscerated, a small piece of tissue (approximately 2 g) was removed from the right pectoral muscle using a scalpel. The tissue was then carefully placed in a 2 mL cryogenic vial and labeled with the sample number. To ensure consistency, we attempted to maintain similar weights within each group as much as possible, despite variations in individual chicken weights. The samples were rapidly frozen in liquid nitrogen for 15 min and transported on dry ice to Sichuan PANOMIX Biotech Co., Ltd. (Chengdu, China). The muscle tissues were analyzed quantitatively and qualitatively for fatty acid composition using gas chromatography–mass spectrometry (24, 25), and amino acid content was analyzed using ultrahigh-performance liquid chromatography-mass spectrometry (26, 27).

### 2.5 Gut microbiome analysis of 16S gene

Extraction of cecum contents was performed as described previously (28). The VAHTSTM DNA Clean Beads Kit (Nanjing Vazyme Biotech Co., Ltd., Nanjing, China) was used to extract total DNA from the cecal contents of the broilers according to the manufacturer’s instructions. The V3–V4 variable regions of the 16S rRNA gene in the microbial community were then amplified from the DNA through PCR using a forward (5′-ACTCCTACGGGAGGCAGCA-3′) and reverse (5′-GGACTACHVGGGTWTCTAAT-3′) primer. The products were quantified fluorometrically using a Quant-iT PicoGreen dsDNA Assay Kit and a microplate reader (BioTek, FLx800). Based on the fluorometric quantification results, the samples were pooled in their respective proportions to meet the sequencing requirements. Sequencing libraries were prepared using the TruSeq Nano DNA LT Library Prep Kit (Illumina, San Diego, CA, United States). Sequencing was performed on an Illumina platform (Sichuan PANOMIX Biotech Co., Ltd., Chengdu, China). The resulting 16S rRNA sequence data were submitted to the National Center for Biotechnology Information’s NCBI Sequence Read Archive database (Bethesda, MD, United States). The accession numbers range from SRA: SRR26286147 to SRR26286161.

### 2.6 Statistical analysis

Microsoft Office Excel 2021 and GraphPad Prism 10.0.3 were used to record, analyze, and visualize the data. Statistical analyses were conducted on the cloud-based SPSSAU platform (Statistical Product and Service Software Automatically; https://spssau.com/). The analyses included one-way analysis of variance (ANOVA) and Tukey’s post-hoc multiple comparison tests. The Kruskal–Wallis test was used to compare the alpha diversity of the gut microbiota between groups, followed by Dunn’s post-hoc test. Furthermore, to investigate the interrelation between diverse parameters and gut microbiota abundance at the genus level, and to eliminate the effects of unit variations at the numerical scale, we used the Sum Normalization approach with the formula X/Sum(X) for dimensionless data handling. Subsequently, we conducted correlation analyses using the PANOMIX cloud analysis platform, available at https://www.biodeep.cn/. We used version 3.6.3 of the R software package to analyze and visualize the 16S gut microbiota dataset, excluding alpha diversity. Beta diversity was depicted using a principal coordinates analysis (PCoA) plot.

## 3 Results

### 3.1 Growth performance

In a preliminary exploration of the effects of *L. cubeba* fruit extract on the growth performance of white-feather broilers, no significant (*p* > 0.05) differences were observed in the final body weight, ADG, ADFI, or F/G among the three groups of broilers during the early (7–21 d) or late (22–42 d) phases of the experiment. These results indicate that the inclusion of *L. cubeba* extract in the diet of broilers had no significant impact on their growth performance (Table 2).

**Table 2:** Effects of the *L. cubeba* extract on growth performance of white-feather broilers.

Items	Treatments			*F*	*p*
	CON (*n* = 16)	L (*n* = 16)	H (*n* = 16)		
**7–21 days (early stage)**					
Initial weight (g)	82.49 ± 2.25	85.02 ± 2.17	79.31 ± 3.27	1.202	0.310
Final weight (g)	430.70 ± 16.76	437.70 ± 15.13	417.20 ± 13.82	0.465	0.631
ADG (g)	16.58 ± 0.73	16.79 ± 0.68	16.09 ± 0.55	0.302	0.741
ADFI (g/day)	43.13 ± 3.43	43.30 ± 2.97	42.47 ± 3.06	0.019	0.980
F/G	2.68 ± 0.12	2.64 ± 0.10	2.69 ± 0.09	0.056	0.946
**22–42 days (late stage)**					
Initial weight (g)	430.70 ± 16.76	437.70 ± 15.13	417.20 ± 13.82	0.465	0.631
Final weight (g)	1524.00 ± 88.46	1437.00 ± 52.31	1527.00 ± 50.73	0.795	0.460
ADG (g)	51.45 ± 3.55	47.92 ± 2.36	52.88 ± 1.73	1.269	0.295
ADFI (g/day)	116.60 ± 3.36	120.30 ± 2.80	126.70 ± 4.66	1.915	0.150
F/G	2.38 ± 0.24	2.61 ± 0.15	2.43 ± 0.08	0.624	0.542
**7–42 days (full experiment duration)**					
Initial weight (g)	82.49 ± 2.25	85.02 ± 2.17	79.31 ± 3.27	1.202	0.310
Final weight (g)	1524.00 ± 88.46	1437.00 ± 52.31	1527.00 ± 50.73	0.795	0.460
ADG (g)	34.31 ± 2.08	32.20 ± 1.26	34.52 ± 1.14	0.921	0.409
ADFI (g/day)	100.70 ± 3.82	103.60 ± 3.62	108.40 ± 4.81	0.906	0.405
F/G	3.04 ± 0.26	3.28 ± 0.13	3.18 ± 0.10	0.547	0.584

Differences between means are not statistically significant (*p* > 0.05). Data are presented as mean ± standard error of the mean (SEM). CON, control group (no *L. cubeba* supplementation); L, low-dose group (1.25 g/kg of *L. cubeba* diet supplementation); H, high-dose group (2.5 g/kg of *L. cubeba* supplementation). ADG, average daily gain; ADFI, Average daily feed intake; feed-to-gain ratio (F/G).

### 3.2 Slaughter performance

**Figure 2:**
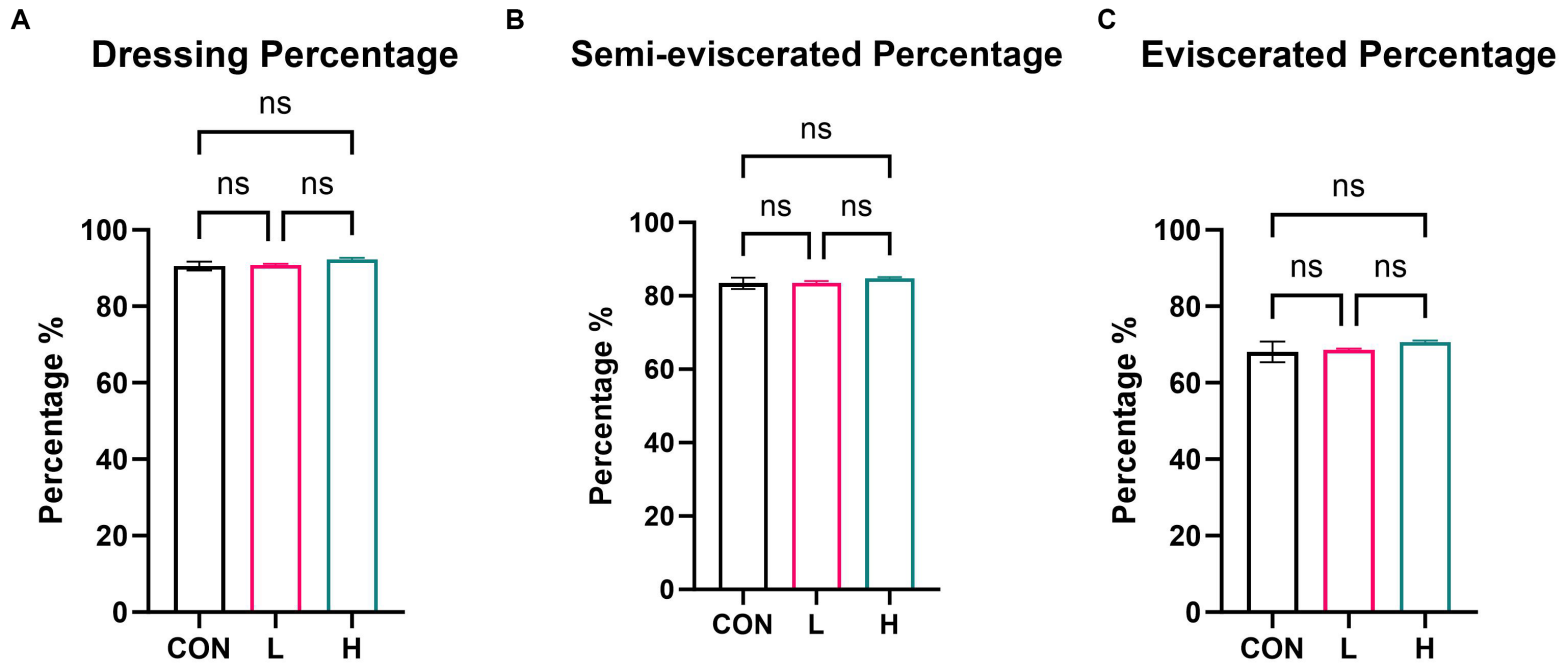
Effects of *L. cubeba* fruit extract on white-feather broiler slaughter performance metrics: **(A)** dressing percentage, **(B)** semi-eviscerated percentage, and **(C)** eviscerated percentage. CON, control group (no *L. cubeba* supplementation); L, low-dose group (1.25 g/kg of *L. cubeba* diet supplementation); H, high-dose group (2.5 g/kg of *L. cubeba* supplementation). ns, not significant (*p* > 0.05).

On day 42, five broilers in each group were randomly slaughtered. Compared to the control group, neither the low- nor high-dose groups significantly affected dressing, semi-evisceration, or evisceration percentages (*p* > 0.05) (Figure 2). The inclusion of *L. cubeba* extract in the daily diet of broilers had no impact on their slaughter performance.

### 3.3 Meat quality

**Figure 3:**
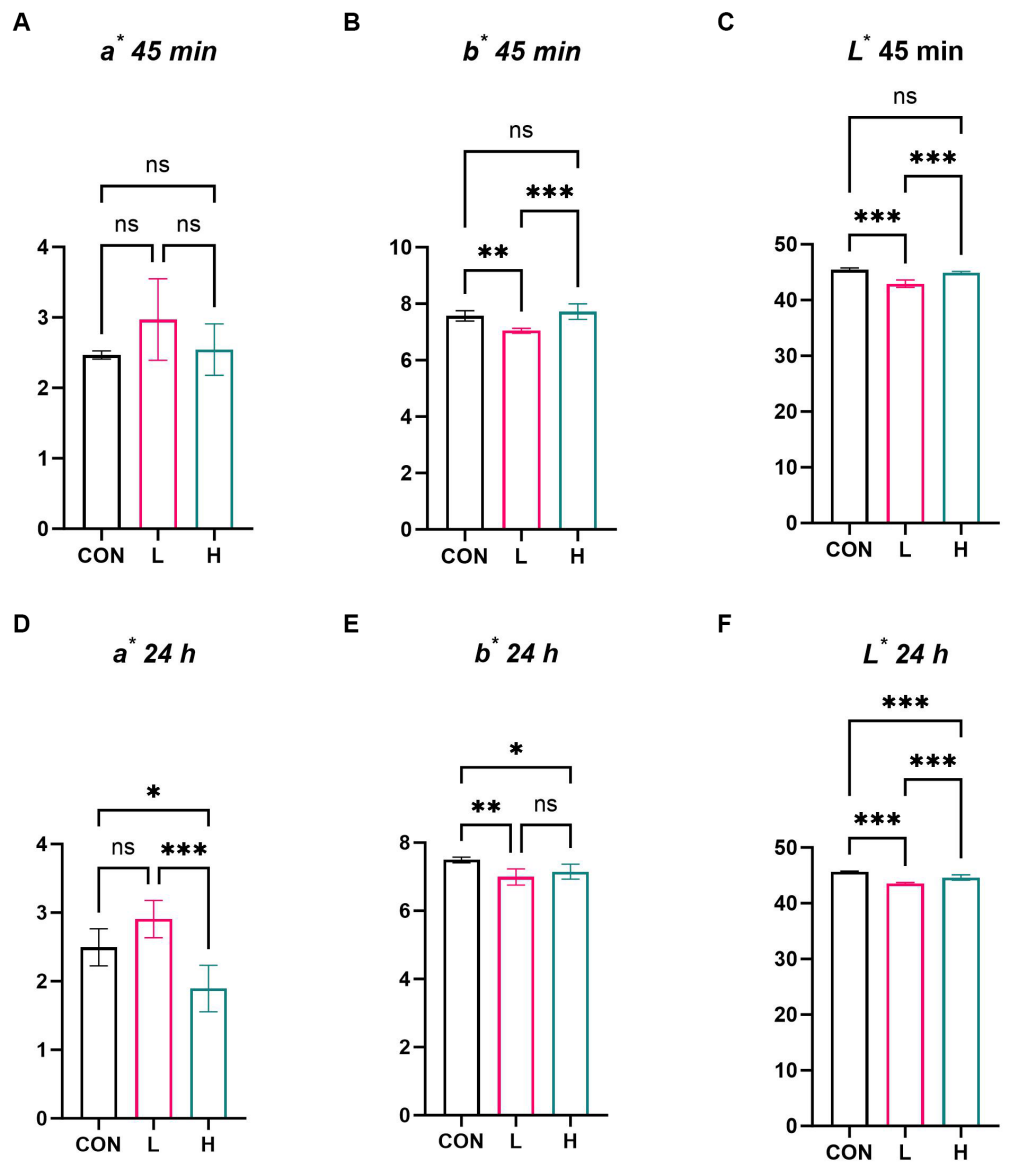
Effects of *L. cubeba* fruit extract on coloration of white-feather broiler breast meat. **(A–C)** Differences in the **(A)** a^*^ value, **(B)** b^*^ value, **(C)** and L^*^ value at 45 min after slaughter. **(D–F)** Differences in the **(D)** a^*^ value, **(E)** b^*^ value, and **(F)** L^*^ value 24 h after slaughter. ns, not significant (*p* > 0.05), ^*^p < 0.05, ^**^p < 0.01, and ^***^p < 0.001. a^*^, redness; b^*^, yellowness; L^*^, luminance.

In the evaluation of the physical parameters of chicken breast meat, the a^*^ values of the chicken breasts showed no significant differences among the dose groups at 45 min (Figure 3A); however, there was a noticeable difference in the b^*^ and L^*^ values at this time point (*p* < 0.01), although clear trends were observed based on dose (Figures 3B,C). After 24 h of re-evaluation, differences were observed in a^*^, b^*^ (Figures 3D,E), and L^*^ values (Figure 3F) (*p* < 0.01), meaning that the meat color of the chicken breasts exhibited significant changes after 24 h. Multiple comparisons revealed a dose-dependent decrease in both a^*^ and b^*^ values at 24 h after slaughter.

**Figure 4:**
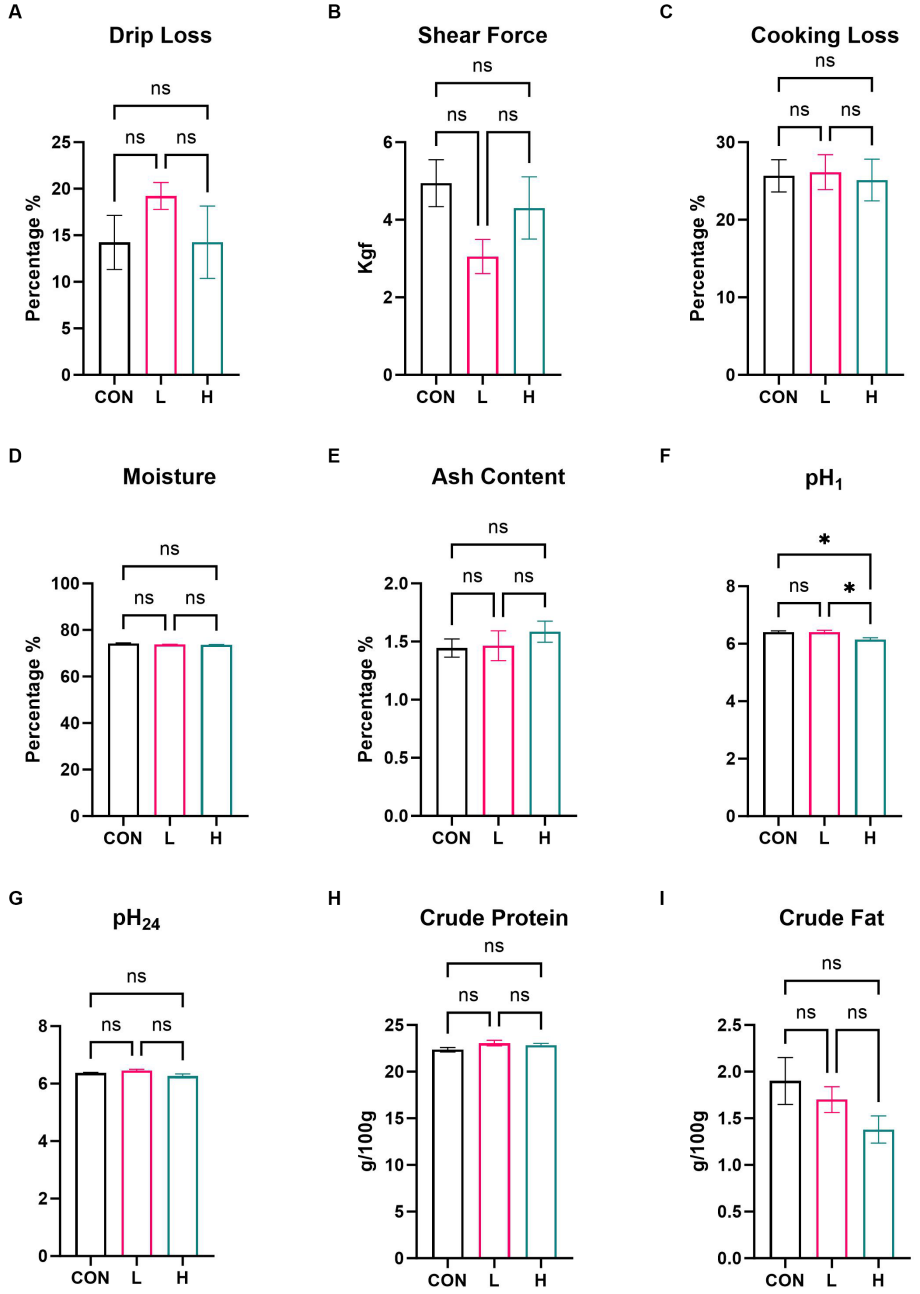
Effects of supplementation with *L. cubeba* fruit extract on broiler chicken breast meat. **(A)** Drip loss. **(B)** Shear force. **(C)** Cooking loss. **(D)** Moisture. **(E)** Ash content. **(F)** pH value at 45 min post-mortem. **(G)** pH value at 24 h post-mortem. **(H)** Crude protein content. **(I)** Crude fat content. ns, not significant (*p* > 0.05); ^*^p < 0.05.

In the comprehensive assessment of meat quality, there were no significant differences in measurements of multiple indicators, such as drip loss, shear force, cooking loss, moisture, ash content (Figures 4A–E), pH_24_, crude protein, and crude fat (Figures 4G–I), among the three groups of broilers. Measurement of pH_1_ (Figure 4F) revealed a decrease in pH level in the group administered high doses compared to that in the control group (*p* = 0.009), suggesting that supplementation with *L. cubeba* extract in the diet can influence the pH of the breast meat post slaughter.

### 3.4 Profile of amino acids

**Figure 5:**
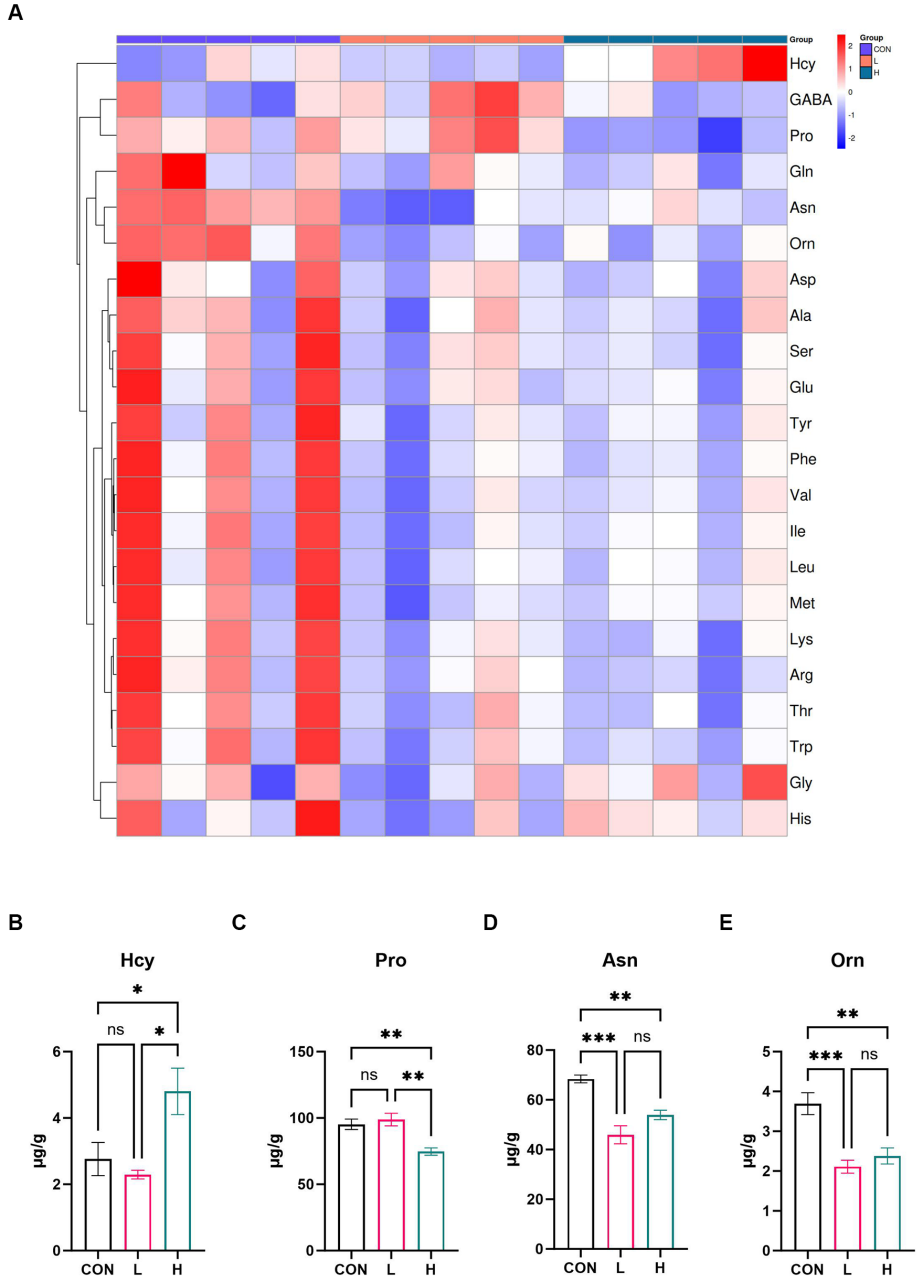
Effects of *L. cubeba* fruit extract on amino acid levels in white-feather broiler breast meat. **(A)** Heatmap comparing the amino acid levels in the low-dose (L; 1.25 g/kg of *L. cubeba*), high-dose (H; 2.5 g/kg of *L. cubeba*), and control (CON) groups. **(B–E)** Relative contents of **(B)** Hcy, **(C)** Pro, **(D)** Asn, and **(E)** Orn. ns, not significant (*p* > 0.05); ^*^p < 0.05, ^**^p < 0.01, and ^***^p < 0.001. The amino acid abbreviations in the image correspond to these full names: Hcy (Homocysteine), GABA (Gamma-aminobutyric acid), Pro (Proline), Gln (Glutamine), Asn (Asparagine), Orn (Ornithine), Asp (Aspartic acid), Ala (Alanine), Ser (Serine), Glu (Glutamic acid), Tyr (Tyrosine), Phe (Phenylalanine), Val (Valine), Ile (Isoleucine), Leu (Leucine), Met (Methionine), Lys (Lysine), Arg (Arginine), Thr (Threonine), Trp (Tryptophan), Gly (Glycine), and His (Histidine).

A targeted amino acid metabolomic analysis was conducted to further investigate the amino acid composition of the broiler breast muscle. As shown in the heatmap (Figure 5A), there were distinct differences in the relative content of various amino acids among the control, low-, and high-dose groups. Specifically, there was a greater amount of certain amino acids in the high-dose group than in the low-dose group, as detailed in Supplementary Table S3. The bar charts (Figures 5B–E) further delineate the absolute content differences of four amino acids (Hcy, Pro, Asn, and Orn) across different groups (*p* < 0.01). Hcy levels were significantly higher in the high-dose group than in the control (*p* = 0.04) or low-dose groups (*p* = 0.01). Pro levels were significantly lower in the high-dose group than in the control (*p* = 0.007) and low-dose groups (*p* = 0.002). Asn concentrations in the high-dose (*p =* 0.004) and low-dose groups (*p* < 0.001) were substantially lower than those in the control group. Orn levels decreased significantly in the high-dose group (*p =* 0.003) and even more so in the low-dose group (*p <* 0.001) when compared with those in the control group. These findings suggest that supplementation with *L. cubeba* extract markedly influences the amino acid composition of breast muscle in the diet of broiler chickens.

### 3.5 Profile of fatty acids

**Figure 6:**
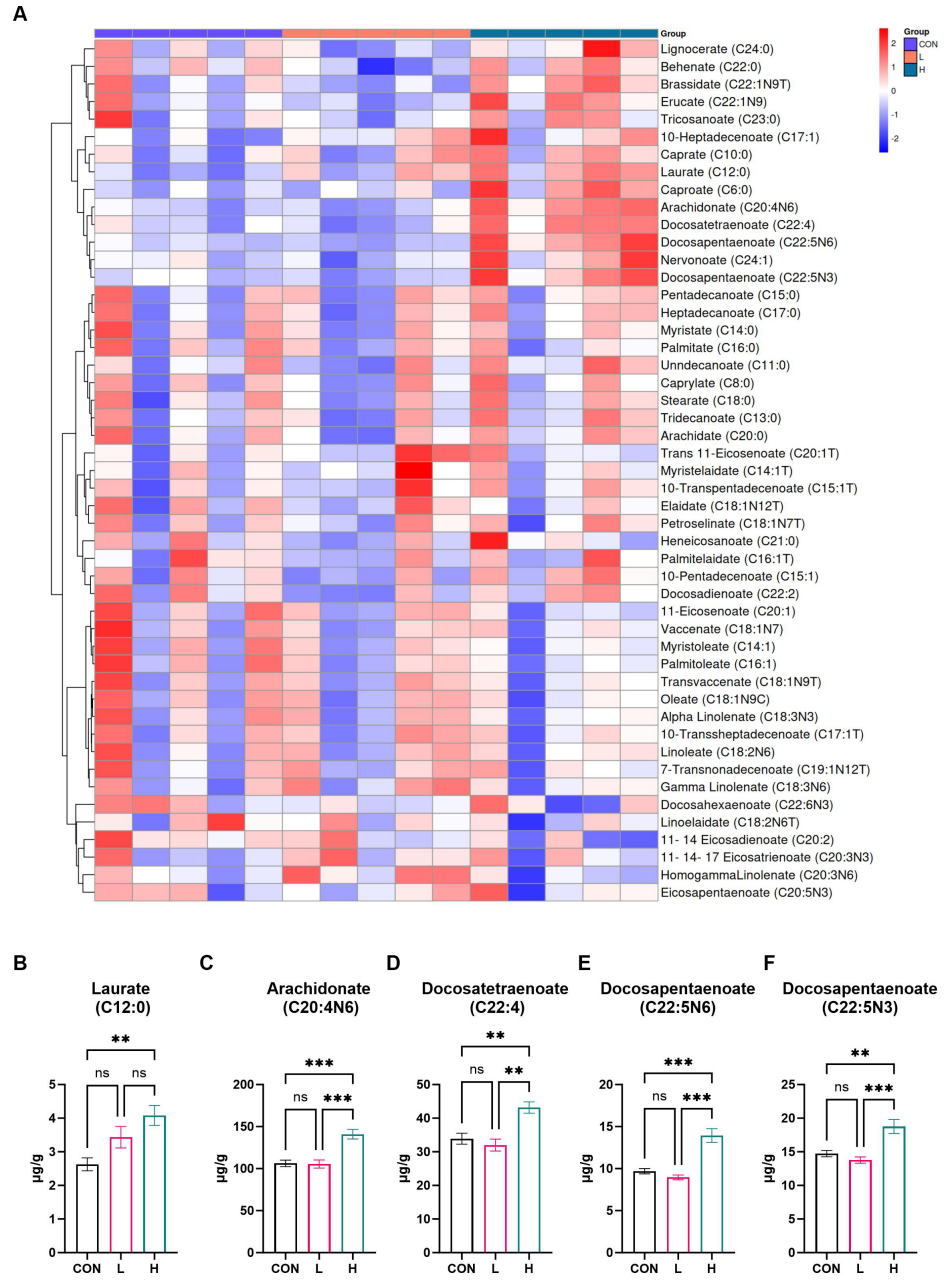
Effects of *L. cubeba* fruit extract on fatty acid levels in broiler chicken breast meat. **(A)** Heatmap comparing the fatty acid profiles of the low-dose (L; 1.25 g/kg *L. cubeba*), high-dose (H; 2.5 g/kg *L. cubeba*), and control (CON) groups. **(B–F)** Relative contents of **(B)** laurate (C12:0), **(C)** arachidonate (C20:4n6), **(D)** docosatetraenoate (C22:4), **(E)** docosapentaenoate (C22:5n6), and **(F)** docosapentaenoate (C22:5n3). ns, not significant (*p* > 0.05); ^**^p < 0.01 and ^***^p < 0.001.

To examine the fatty acid composition of white-feather broiler breast muscle, a targeted metabolomic analysis was conducted, resulting in the identification of 49 fatty acids. For a detailed list of the identified fatty acids, refer to Supplementary Table S4. Notable differences in fatty acid levels between the high- and low-dose groups and the control group are displayed in the heatmap presented in Figure 6A. The bar chart illustrates the levels of five different fatty acids in chicken breast, varying according to dosage. As shown in Figures 6D–F, the meat contained three unsaturated C22 fatty acids: docosatetraenoate (C22:4), which had a significant presence (*p =* 0.001); its structural variant docosapentaenoate (C22:5n6; *p <* 0.001); and another form of docosapentaenoate (C22:5n3; *p <* 0.001). Furthermore, arachidonate (C20:4n6), an unsaturated C20 fatty acid, exhibited the highest absolute increase compared to that in the control group (*p <* 0.001) (Figure 6C). Laurate (C12:0), a type of saturated fatty acid, exhibited the smallest absolute increase compared to that in the control group (*p <* 0.001) (Figure 6B). This suggests that *L. cubeba* extract has a significant impact on the levels of unsaturated fatty acids in chicken breasts, dependent on the amount of extract administered.

### 3.6 Intestinal microbiome analysis

**Figure 7:**
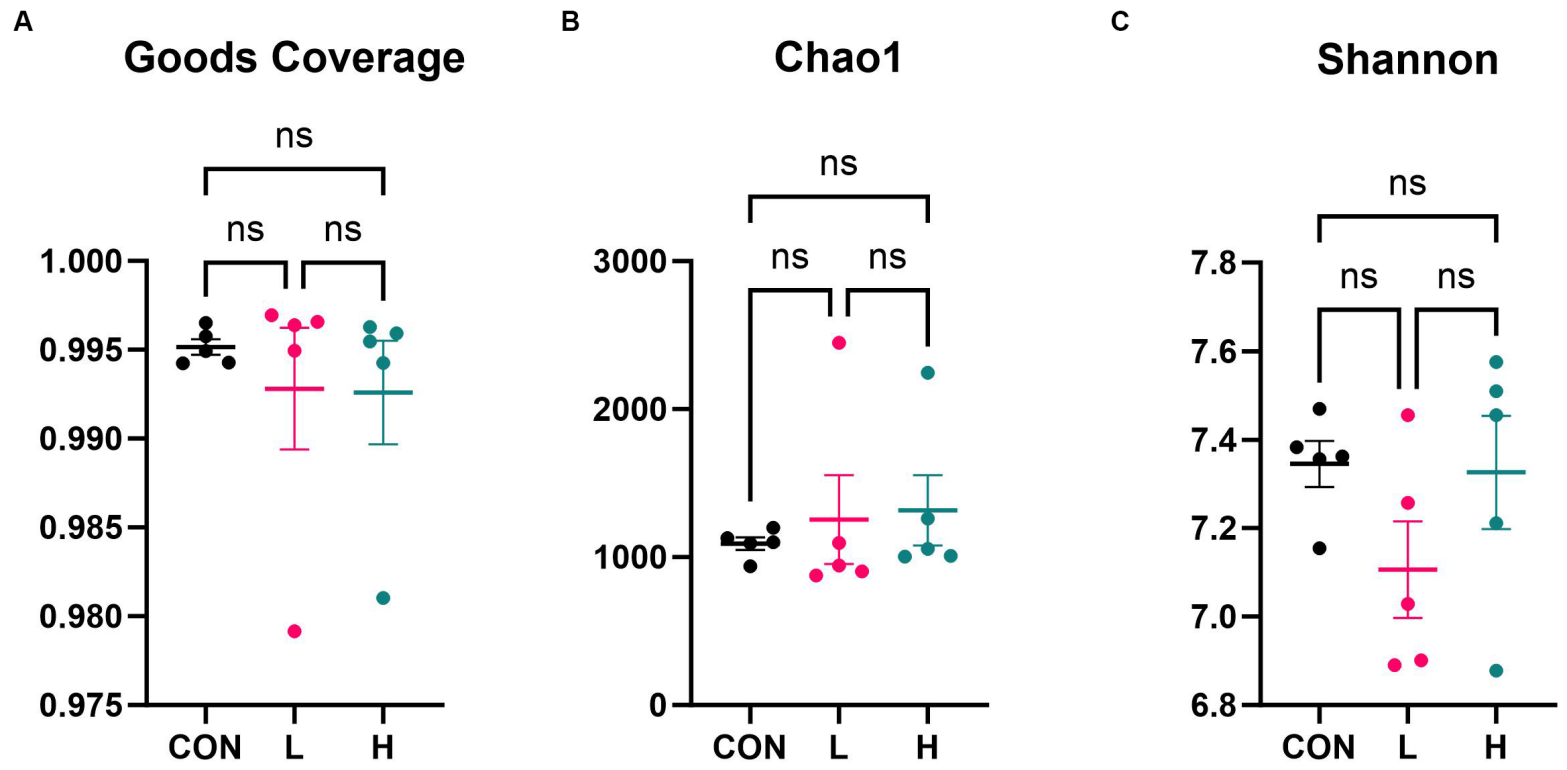
Alpha diversity indices of the cecum flora. **(A)** Goods coverage, **(B)** Chao1, and **(C)** Shannon diversity indices. CON, control group (no *L. cubeba* supplementation); L, low-dose group (1.25 g/kg of *L. cubeba* supplementation); H, high-dose group (2.5 g/kg of *L. cubeba* supplementation). ns, not significant (*p* > 0.05).

Alpha diversity analysis based on 16S rRNA gene sequencing revealed that there were no significant differences in microfloral diversity among the three groups. The Goods Coverage index (Figure 7A), with mean values approaching one, indicated that the sequencing depth was sufficient to cover most of the microbial community. Species richness, estimated using the Chao1 index (Figure 7B), showed no significant difference across the groups, with values ranging from 1,000 to 3,000. The Shannon index (Figure 7C), which reflects species richness and evenness, varied between 7.0 and 7.8, but there were no significant differences among the groups. These three indices indicated no significant differences in the microbial alpha diversity among the three groups, but there may have been subtle variations in community structure and species abundance.

**Figure 8:**
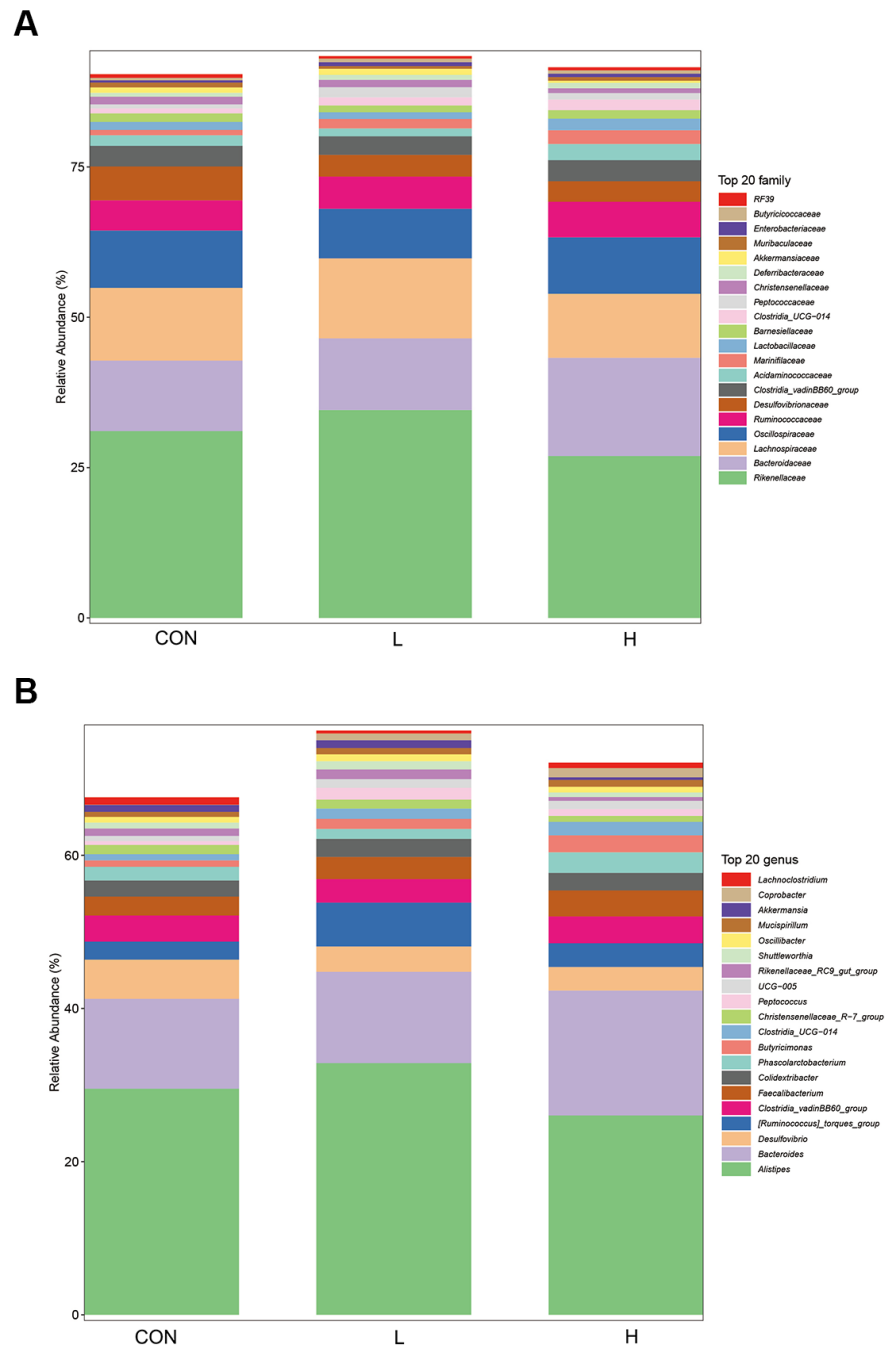
Composition of gut bacterial species at the family **(A)** and genus **(B)** levels in the control (CON), low-dose (1.25 g/kg of *L. cubeba*), and high-dose (H; 2.5 g/kg of *L. cubeba*) groups.

Analysis of family level 16S rRNA gene sequencing revealed significant differences in the microbial community structures among the control, low-dose, and high-dose groups. As shown in Figure 8, certain microbes at the family level (Figure 8A), such as Muribaculaceae and Lachnospiraceae, showed varying relative abundances among the dosage groups. At the genus level (Figure 8B), microbial genera, such as *Lachnoclostridium* and *Coprobacter,* also exhibited differences in their relative abundances across the three treatments. These findings suggest that *L. cubeba* extract may influence the composition and structure of gut microbial communities.

**Figure 9:**
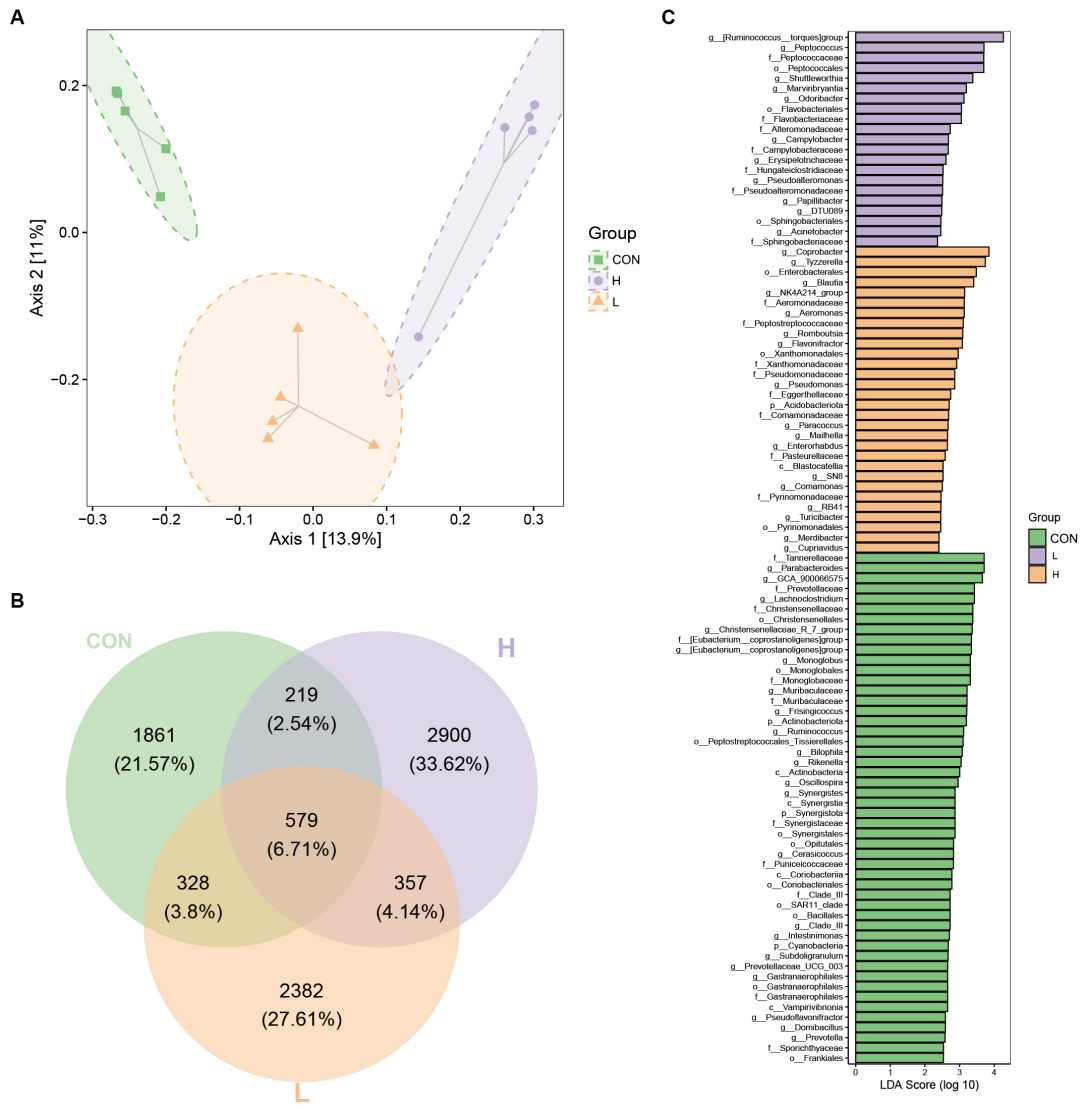
Beta diversity indices of gut bacterial species among groups. **(A)** Two-dimensional ordination plot of samples using Jaccard-based principal components analysis (PCoA) with Ellipse for the control (CON), low-dose (L; 1.25 g/kg of *L. cubeba*), and high-dose (H; 2.5 g/kg of *L. cubeba*) groups. **(B)** Venn diagram representation of amplicon sequence variants/operational taxonomic units in the samples from each group. **(C)** Bar chart of linear discriminant analysis (LDA) effect values for indicator species.

Beta diversity analysis based on 16S rRNA gene sequencing, as shown in the PCoA plot, revealed a significant separation between the three dose treatment groups, with axes 1 and 2 accounting for 13.9% and 11% of the variance, respectively (Figure 9A). The PCoA plot shows a significant separation between the three dose treatment groups, with axes 1 and 2 accounting for 13.9 and 11% of the variance, respectively. A Venn diagram (Figure 9B) shows the number of unique and shared operational taxonomic units (OTUs) between the groups. Specifically, the groups were divided into a control, high-dose, and low-dose group, which contained 1861 (21.57%), 2,900 (33.62%), and 2,382 (27.61%) unique OTUs, respectively. Furthermore, 579 OTUs (6.71%) were shared among the three treatments. Linear discriminant analysis (LDA; Figure 9C) revealed significant differences in microbial taxa between treatments. Among these, differential taxa were more abundant in the control group. The top two genera based on LDA scores were *Parabacteroides* (LDA score = 3.72) and *GCA_900066575* (LDA score = 3.66) in the control group, *Ruminococcus__torques_group* (LDA score = 4.26) and *Peptococcus* (LDA score = 3.71) in the low-dose group, and *Coprobacter* (LDA score = 3.85) and *Tyzzerella* (LDA score = 3.74) in the high-dose group.

**Figure 10:**
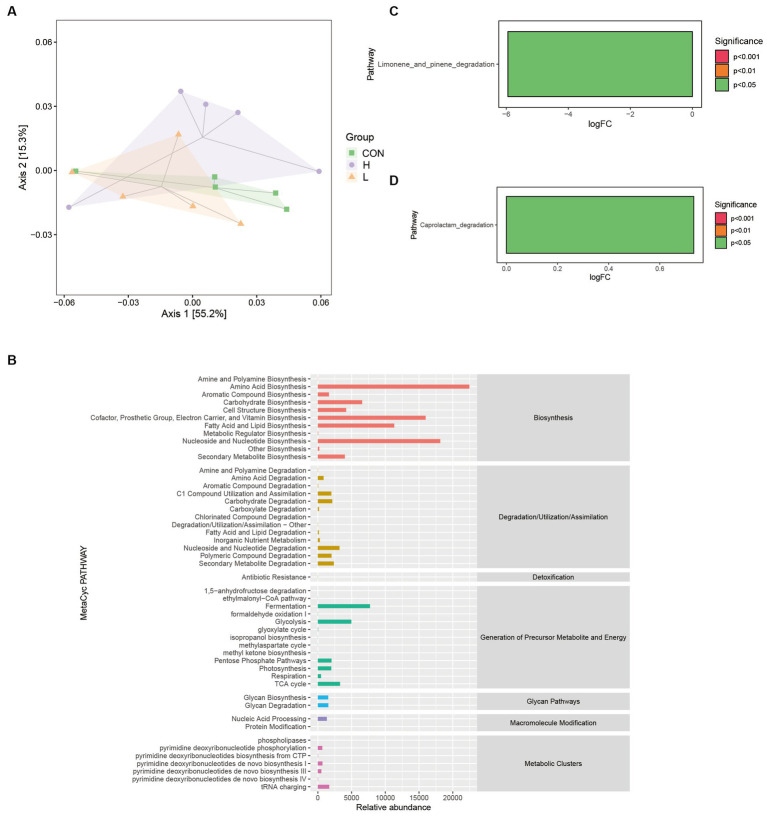
Differences in the Kyoto Encyclopedia of Genes and Genomes (KEGG) metabolic pathways among groups. **(A)** Bray–Curtis-based PCoA for functional units with hulls. **(B)** Predicted abundance profiles of the MetaCyc secondary functional pathways. **(C)** Differential analysis of KEGG metabolic pathways between the control (CON) and low-dose (L; 1.25 g/kg *L. cubeba*) groups. **(D)** Differential analysis of KEGG metabolic pathways between the CON and high-dose (H; 2.5 g/kg *L. cubeba*) groups.

The number of functional units (KOs) was large, and PCoA was used to clarify the functional differences of the samples in reduced dimensions. Figure 10A reveals that the first two coordinates explained 70.5% of the overall variation. Coordinate 1 contributed 55.2%, whereas coordinate 2 contributed 15.3%. The analysis revealed that the line plots of the 16S gut bacterial composition of the broilers in the three dose groups overlapped, indicating limited discrimination of the three groups by the two coordinates. Figure 10B shows the potential metabolic pathways associated with microbial communities by statistically enriching the abundance of secondary functional pathways from the MetaCyc database. These pathways are involved in the biosynthesis of amino acids (relative abundance = 22,458) and fatty acids and lipids (relative abundance = 11,324.9). Figures 10C,D highlight the primary functional differences based on Kyoto Encyclopedia of Genes and Genomes (KEGG) enrichment (*p* < 0.05) among the three groups. The low-dose group primarily involved the limonene and pinene degradation metabolic pathways, whereas the high-dose group primarily involved the caprolactam degradation metabolic pathway.

**Figure 11:**
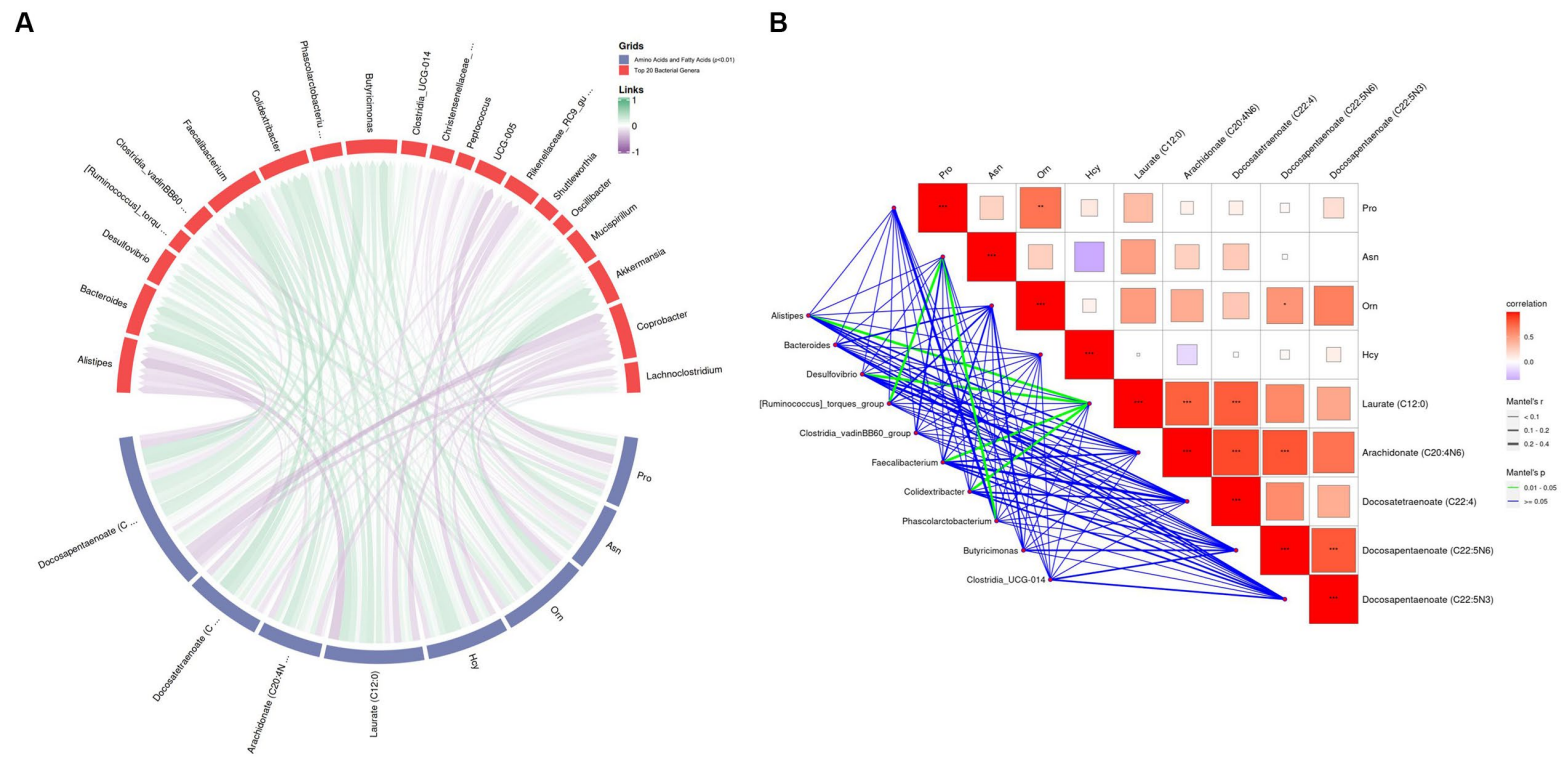
Correlation analysis between amino acids and polyunsaturated fatty acids at *p* < 0.01 with the top 20 genus-level gut flora. **(A)** Chord diagram of the top 20 genus-level intestinal flora with amino acid and fatty acid correlation. **(B)** Mantel test between top 20 genus-level intestinal flora, amino acids, and fatty acids. In the collinearity analysis, Variable 1 comprised the top 20 abundant bacteria, while Variable 2 comprised four amino acids and five fatty acids.

The correlation analysis revealed that, at the genus level, there was a positive correlation between *Alistipes* (*r* = 0.235, *p* = 0.033), *Desulfovibrio* (*r* = 0.320, *p* = 0.014), *Faecalibacterium* (*r* = 0.232, *p* = 0.036), *Colidextribacter* (*r* = 0.354, *p* = 0.013), and laurate (C12:0; Figures 11A,B). Laurate (C12:0) was correlated with arachidonate (C20:4n6; *r* = 0.849, *p* < 0.001) and docosatetraenoate (C22:4; *r* = 0.862, *p* < 0.001) (Figure 11A). *Alistipes* (*r* = 0.232, *p* = 0.036) and *Phascolarctobacterium* (*r* = 0.303, *p* = 0.034) were positively correlated with Asn levels (Figures 11A,B). These results suggest that *L. cubeba* extract in the broilers’ diets modulate the gut microbiota at the genus level, particularly *Alistipes*, *Desulfovibrio*, *Faecalibacterium*, and *Colidextribacter*, which indirectly affect docosatetraenoate (C22:4) and arachidonate (C20:4n6) levels via laurate (C12:0).

## 4 Discussion

In 2022, China was one of the top five global destinations for broiler chicken exports, particularly the white-feather broiler because of its rapid growth rate, efficient feed conversion ratio, and high muscle protein content, garnering considerable favor in the international market (2, 29). In the post-antibiotic era, feed additives have become increasingly important for improving animal productivity, maintaining animal health, lowering feed costs, and improving the quality of livestock products (30).

This study aimed to explore the potential value of *L. cubeba* extract as a feed additive in the rearing of broilers. By incorporating various doses of *L. cubeba* extract into the basic broiler diet, this study systematically assessed the impact of this additive on the growth and slaughter performance of white-feather broilers. Furthermore, this research delved into the potential effects of *L. cubeba* extract on the sensory and nutritional quality of the broiler’s breast meat. To comprehensively evaluate the efficacy of this additive, we discuss its effects from three dimensions: growth performance, slaughter performance, and meat quality. Through this multi-faceted evaluation, the study aimed to provide a scientific basis for using *L. cubeba* extract in livestock farming.

In the experiment, either 1.25 or 2.50 g/kg of *L. cubeba* fruit extract was added to broiler diet. The results indicated that at these two dose levels, *L. cubeba* fruit extract did not significantly impact the growth performance of broilers. Notably, in similar studies where quercetin was added to the diet, although its effect on enhancing the growth performance of broilers was not significant, it was observed to positively influence the maintenance of the intestinal microecological balance, and it also reduced mortality (31). These findings suggest that while such additives might be beneficial for poultry growth under certain conditions, their role in enhancing the growth performance of broilers appears to be limited. It is worth mentioning that this study involved multiple key indicators to evaluate the growth performance of broilers, including body weight, ADFI, ADG, and F/G. These indicators are commonly used to comprehensively assess the growth performance of broilers and are important for understanding and improving the efficiency of broiler production (32). The statistically insignificant differences observed in this study may stem from certain limitations in the experimental design, particularly the small sample size and relatively large standard error within the experimental groups. This underscores the importance of appropriately increasing sample sizes in future research to ensure sufficient statistical power.

In evaluating the slaughter performance of broilers, this study primarily focused on three key indicators: dressing percentage, semi-eviscerated percentage, and eviscerated percentage. It is noteworthy that these indicators did not exhibit significant differences among the experimental groups. Additionally, as these indicators were calculated using body weight as the denominator, they effectively minimized the impact of weight factors on the assessment of slaughter performance. It is important to note that in dietary supplementation experiments on broilers, capsaicin has induced minor, but significant, differences in growth performance compared to quercetin; however, no significant enhancement effect of capsaicin on slaughter performance has been observed (23). The findings of the present study suggest that under the specific conditions of this experiment, the observed parameters were influenced by certain unforeseen factors, such as breed advantages of the broilers and variables in the rearing environment. Moreover, this outcome indicates that the limited nature of the dosing method or sample size used in the experimental design may have led to a lack of sufficient differences in these indicators. Therefore, further research needs to consider these potential variables and may require a broader sample size and different dosing methods, such as water medication, to ensure the accuracy and reliability of the results.

In this article, we focused on the meat quality of broilers, particularly addressing two aspects: sensory quality and nutritional value. This study revealed that adding *L. cubeba* fruit extract to the basic diet of broilers significantly enhanced the coloration of chicken breast meat, especially in reducing its L^*^ value (lightness). This change is closely linked to the overall improvement in the sensory meat quality of broiler breast meat (33).

Our research showed a significant difference in pH values at 45 min for chicken breast samples from the high-dose extract group compared with those of the control group. However, no significant difference was observed for the 24 h pH measurement. In terms of nutritional value, the study found no significant difference in the content of crude protein and crude fat. To further investigate the nutritional value of chicken breast, GC-MS and UPLC-MS were used to measure fatty acids and amino acids, respectively. The results showed that adding *L. cubeba* fruit extract increased the content of homocysteine in chicken breast muscles. The interaction of cysteine and ribose in chicken breast through the Maillard reaction, commonly used to study the mechanism of generating meat flavor (34), is especially pertinent under environmental or high temperatures (35). The chemical reactions occurring in fried breast meat are key factors in forming its unique aroma and flavor characteristics, leading to the generation of specific flavor substances (36). In terms of fatty acids, a dose-dependent increase in the content of unsaturated fatty acids C22 and C20 was noted. After adding *L. cubeba* fruit extract to the basic diet, the contents of arachidonic acid (C20:4n6), docosapentaenoic acid isomers (C22:5n6 and C22:5n3), and docosatetraenoic acid (C22:4) significantly increased. Arachidonic acid (C20:4n6) has been shown to play a crucial role in avian nutrition by affecting prostaglandin production, inflammatory responses, blood coagulation, and immune functions (37). Takahashi (38) confirmed that supplementing diets with arachidonic acid and considering polymorphisms of FADS1 and FADS2 as genetic selection markers can effectively regulate arachidonic acid content in chicken meat, thus improving the texture and flavor of the meat. Meanwhile, the oxidation of fatty acids in chicken breast muscle simultaneously provides specific flavor characteristics and, during the heating process, combines with amino acids in proteins and other components to become precursors to flavor substances (34).

As white-feathered broilers are not model organisms, and because they exhibit a hybrid lineage with considerable genetic variability, exploring the molecular biological mechanisms at the transcriptional level poses challenges. However, genetic analysis of the gut microbiota at the genus level (16S rRNA) in this study revealed that the intake of *L. cubeba* by broilers positively modulated specific gut microorganisms, including members of the genera *Alistipes*, *Desulfovibrio*, *Faecalibacterium*, and *Colidextribacter*. This modulation occurred indirectly through the concentration of laurate (C12:0), which positively affected the concentrations of docosatetraenoate (C22:4) and arachidonate (C20:4n6). However, due to the lack of research on beneficial poultry gut microbes, future studies should explore whether the relative abundances of *Alistipes*, *Desulfovibrio*, *Faecalibacterium*, and *Colidextribacter* correlate with healthy poultry growth.

Research has revealed complex regulatory mechanisms by which plants and their extracts exert their effects on animal systems (39). The bioactivity of medicinal plants is significantly affected by the methods used for their processing and extraction (40). Therefore, it is important to progress beyond analyses focused on specific compounds or classes of compounds to elucidate plant functions. The effects of plants on animals, especially poultry, also manifest this intricate mechanistic complexity (41). Adopting Mendelian randomization as a methodology is crucial for analyzing these cause-effect relationships more objectively (42), as this method employs genetic variation as an instrumental variable to evaluate the causal relationships between a particular influence and a specific outcome, making it highly applicable to broiler chicken production. Using Mendelian randomization, we analyzed the relationship between the nutrition and health of white-feather broilers, discovering genetic variations linked to poultry nutrition and meat quality that will determine the actual consequences of a specific feed additive on production efficiency or disease incidence. Furthermore, consideration of the differences in drug metabolism between poultry and humans, including drug absorption, distribution, and excretion within the body, is critical, as some medications may not have the same effects in humans as in poultry. Therefore, more in-depth investigations are needed on these differences to verify the possible benefits of herbal feed additives.

In summary, this study indicates that fruit extract additives of *L. cubeba* have the potential to serve as sustainable feed additives, replacing antibiotics and chemically synthesized drugs. Use of these additives can have a positive influence on the coloration of white-feather broiler breast meat by adjusting the post-slaughter meat pH, flavor-associated amino acids, and the content of certain unsaturated fatty acids. However, in this study, we intended to obtain preliminary observations and results; therefore, only a small sample size was used for this pilot investigation. Subsequent research will require a larger sample size and should be extended to a larger population. Despite these limitations, our findings revealed a significant increase in the content of three unsaturated fatty acids: docosatetraenoate (C22:4) (*p* = 0.001), docosapentaenoate (C22:5n6) (*p* < 0.001), and docosapentaenoate (C22:5n3) (*p* < 0.001). As commercial broiler chickens are not animal models, it is challenging to delve further into the underlying mechanisms. We plan to explore these associations using 16S rRNA gut microbiota analyses. In this study, correlation analyses indicated that supplementation of white-feather broiler diets with *L. cubeba* extract can alter the gut microbiota at the genus level, and these changes indirectly affected the levels of docosatetraenoate (C22:4) and arachidonate (C20:4n6) through laurate (C12:0).

## 5 Conclusion

This study investigated the potential effects of adding *L. cubeba* fruit extract to the basic diet of broilers. The results indicated that the addition of 1.25 and 2.50 g/kg of the extract had no significant impact on the growth and slaughter performance of white-feather broilers. However, it significantly improved the coloration of the breast meat, thereby enhancing its sensory quality. Additionally, the extract had a positive influence on the content of amino acids and minor unsaturated fatty acids in the breast meat by modulating the gut microbiota, thus enhancing the nutritional value and flavor of the meat. In conclusion, this exploratory study provides preliminary evidence that the addition of *L. cubeba* fruit extract to broiler feed can, at the very least, enhance the taste and nutritional content of white-feather broiler meat.

## Data availability statement

The original contributions presented in the study are publicly available. This data can be found here: https://www.ncbi.nlm.nih.gov/; SRR26286147–SRR26286161.

## Ethics statement

The animal study was approved by Institutional Ethics Committee of Yunnan Agricultural University. The study was conducted in accordance with the local legislation and institutional requirements.

## Author contributions

YLu: Conceptualization, Data curation, Formal analysis, Investigation, Software, Visualization, Writing – original draft, Writing – review & editing. YB: Data curation, Investigation, Writing – review & editing. ZX: Data curation, Investigation, Writing – review & editing. LS: Investigation, Writing – review & editing. JH: Investigation, Writing – review & editing. KW: Data curation, Writing – review & editing. ZZ: Formal analysis, Validation, Writing – review & editing. LihY: Validation, Writing – review & editing. XJ: Formal analysis, Writing – review & editing. JY: Formal analysis, Writing – review & editing. LijY: Formal analysis, Writing – review & editing. RG: Investigation, Writing – review & editing. JW: Investigation, Writing – review & editing. XD: Resources, Supervision, Validation, Writing – review & editing. YLi: Funding acquisition, Writing – review & editing. CF: Conceptualization, Funding acquisition, Methodology, Project administration, Resources, Supervision, Validation, Writing – original draft, Writing – review & editing.
