# Supplementary material for: Exploring possible benefits of Litsea cubeba Pers. extract on growth, meat quality, and gut flora in white-feather broilers
Source: Front Vet Sci. 2024 Jan 11;10:1335208. doi: 10.3389/fvets.2023.1335208 (PMC10823429; doi:10.3389/fvets.2023.1335208)
Supplement: Supplementary file 1 [file Data_Sheet_1.pdf]

# *Supplementary Material*

**Table S1. Effects of the fruit extract of *L. cubeba* on slaughter performance of white feather broilers**

| Items | Treatment | | | *F* | *p* |
|---|---|---|---|---|---|
| | CON(n=5) | L(n=5) | H(n=5) | | |
| Dressing percentage | 90.52 ± 1.16 | 90.79 ± 0.35 | 92.28 ± 0.42 | 3.395 | 0.065 |
| Semi-eviscerated percentage | 83.42 ± 1.56 | 83.54 ± 0.54 | 84.79 ± 0.31 | 1.579 | 0.243 |
| Eviscerated percentage | 68.08 ± 2.68 | 68.63 ± 0.35 | 70.64 ± 0.42 | 2.352 | 0.134 |

The *F*-value and *p*-value are based on one-way analysis of variance followed by Tukey's multiple comparisons test. The parameters labeled with identical superscript letters (a, b) did not exhibit significant differences ($P > 0.05$). Data are presented as Mean ± Std. Error of Mean (SEM).

**Table S2. Effects of the fruit extract of *L. cubeba* on breast muscle quality of white feather broilers**

| Items | Treatment | | | *F* | *p* |
|---|---|---|---|---|---|
| | CON(n=5) | L(n=5) | H(n=5) | | |
| $pH_1$ | $6.41±0.04^a$ | $6.41±0.06^a$ | $6.15±0.06^b$ | 7.232 | 0.009** |
| $pH_{24}$ | 6.37±0.02 | 6.45±0.04 | 6.27±0.07 | 3.357 | 0.070 |
| Shear force/Kgf | 4.94±0.60 | 3.05±0.44 | 4.31±0.80 | 2.297 | 0.143 |
| Drip loss/% | 14.23±2.91 | 19.22±1.45 | 14.25±3.89 | 0.964 | 0.409 |
| Cooking loss/% | 25.66±2.08 | 26.14±2.25 | 25.12±2.69 | 0.047 | 0.955 |
| $a^*$ 45 min | 2.47±0.03 | 2.97±0.26 | 2.54±0.16 | 2.331 | 0.140 |
| $b^*$ 45 min | $7.57±0.08^a$ | $7.04±0.04^b$ | $7.72±0.12^a$ | 16.340 | <0.001*** |
| $L^*$ 45 min | $45.46±0.14^a$ | $42.94±0.29^b$ | $44.88±0.12^{ab}$ | 44.17 | <0.001*** |
| $a^*$ 24 h | $2.49±0.12^{ab}$ | $2.91±0.12^a$ | $1.89±0.15^b$ | 14.86 | <0.001*** |
| $b^*$ 24 h | $7.49±0.04^a$ | $7.00±0.11^b$ | $7.15±0.10^b$ | 8.802 | 0.004** |
| $L^*$ 24 h | $45.64±0.07^a$ | $43.50±0.10^c$ | $44.62±0.22^b$ | 53.85 | <0.001*** |
| Crude protein g/100g | 22.36±0.24 | 23.06±0.29 | 22.84±0.19 | 2.138 | 0.161 |
| Crude fat g/100g | 1.90±0.25 | 1.70±0.14 | 1.38±0.15 | 1.996 | 0.179 |
| Moisture % | 74.23±0.27 | 73.83±0.05 | 73.71±0.07 | 2.702 | 0.146 |
| Ash content % | 1.44±0.08 | 1.46±0.13 | 1.59±0.09 | 0.564 | 0.596 |

The *F*-value and *p*-value are based on one-way analysis of variance followed by Tukey's multiple comparisons test. The parameters labeled with identical superscript letters (a, b) did not exhibit significant differences ($p > 0.05$). Data are presented as Mean ± Std. Error of Mean (SEM).

**Table S3. Effects of *L. cubeba* fruit extract on amino acid levels in white feather broilers breast meat**

| Item | Treatment | | | *F* | *p* |
|---|---|---|---|---|---|
| | CON(n=5) | L(n=5) | H(n=5) | | |
| Gly | 148.47±8.66 | 135.54±7.11 | 153.09±7.69 | 1.343 | 0.298 |
| Ala | 338.87±15.70 | 308.50±11.00 | 305.79±9.53 | 2.21 | 0.152 |
| GABA | 1.03±0.05 | 1.14±0.04 | 1.02±0.02 | 3.192 | 0.077 |
| Ser | 217.18±12.39 | 195.67±6.81 | 190.18±5.36 | 2.671 | 0.110 |
| Pro | 95.24±3.93$^{a}$ | 98.78±4.74$^{a}$ | 74.71±2.72$^{b}$ | 11.198 | 0.002** |
| Val | 132.80±9.99$^{a}$ | 106.62±4.87$^{b}$ | 111.53±3.45$^{ab}$ | 4.286 | 0.039* |
| Thr | 123.17±8.05$^{a}$ | 103.77±5.28$^{ab}$ | 100.06±3.99$^{b}$ | 4.254 | 0.040* |
| Ile | 100.95±8.47$^{a}$ | 79.75±3.71$^{b}$ | 84.75±2.54$^{ab}$ | 4.007 | 0.046* |
| Leu | 202.06±17.69 | 162.82±8.04 | 171.33±5.72 | 3.116 | 0.081 |
| Asn | 68.40±1.56$^{a}$ | 45.95±3.63$^{b}$ | 53.93±1.88$^{b}$ | 20.296 | <0.001*** |
| Orn | 3.69±0.27$^{a}$ | 2.11±0.16$^{b}$ | 2.38±0.20$^{b}$ | 15.076 | <0.001*** |
| Asp | 106.56±9.26 | 94.33±4.14 | 91.63±4.41 | 1.552 | 0.252 |
| Hcy | 2.76±0.50$^{b}$ | 2.29±0.13$^{b}$ | 4.80±0.70$^{a}$ | 7.036 | 0.010** |
| Gln | 155.88±12.97 | 137.49±7.35 | 129.02±5.89 | 2.202 | 0.153 |
| Lys | 204.93±14.24$^{a}$ | 168.74±6.84$^{ab}$ | 161.83±7.46$^{b}$ | 5.266 | 0.023* |
| Glu | 214.32±14.58 | 187.62±6.85 | 187.80±5.83 | 2.412 | 0.132 |
| Met | 79.30±6.52$^{a}$ | 60.93±2.94$^{b}$ | 66.18±1.54$^{ab}$ | 5.019 | 0.026* |
| His | 143.18±10.18 | 123.29±5.46 | 138.45±3.20 | 2.256 | 0.147 |
| Phe | 105.88±8.08$^{a}$ | 85.63±3.71$^{b}$ | 86.77±2.47$^{ab}$ | 4.564 | 0.034* |
| Arg | 135.04±10.31$^{a}$ | 111.93±5.04$^{ab}$ | 102.99±3.65$^{b}$ | 5.658 | 0.019* |
| Tyr | 141.93±9.56 | 123.19±4.37 | 124.85±3.27 | 2.662 | 0.110 |
| Trp | 33.69±2.22$^{a}$ | 28.48±1.31$^{a}$ | 27.95±0.64$^{a}$ | 4.274 | 0.040* |

The *F*-value and *p*-value are based on one-way analysis of variance followed by Tukey's multiple comparisons test. The parameters labeled with identical superscript letters (a, b, c) did not exhibit significant differences ($p > 0.05$). *$p < 0.05$; **$p < 0.01$; ***$p < 0.001$. Data are presented as Mean ± Std. Error of Mean (SEM).

**Table S4. Effects of *L. cubeba* fruit extract on fatty acid levels in white feather broilers breast meat**

| Item | Treatment | | | *F* | *p* |
|---|---|---|---|---|---|
| | CON(n=5) | L(n=5) | H(n=5) | | |
| Caproate (C6:0) | 0.65±0.01$^{b}$ | 0.66±0.02$^{b}$ | 0.74±0.03$^{a}$ | 6.567 | 0.012* |
| Caprylate (C8:0) | 0.55±0.03 | 0.54±0.02 | 0.57±0.02 | 0.397 | 0.681 |
| Caprate (C10:0) | 0.51±0.05 | 0.57±0.06 | 0.64±0.05 | 1.631 | 0.236 |
| Unndecanoate (C11:0) | 0.14±0.00 | 0.14±0.01 | 0.15±0.00 | 1.569 | 0.248 |
| Laurate (C12:0) | 2.63±0.19$^{b}$ | 3.43±0.32$^{ab}$ | 4.08±0.30$^{a}$ | 7.032 | 0.010** |
| Tridecanoate (C13:0) | 0.22±0.01 | 0.21±0.01 | 0.23±0.01 | 0.967 | 0.408 |

| | | | | | |
|---|---|---|---|---|---|
| Myristate (C14:0) | 42.07±4.08 | 39.79±2.93 | 41.94±2.29 | 0.162 | 0.852 |
| Myristelaidate (C14:1T) | 1.54±0.08 | 1.66±0.11 | 1.60±0.07 | 0.448 | 0.649 |
| Myristoleate (C14:1) | 5.70±0.73 | 5.03±0.44 | 4.71±0.39 | 0.871 | 0.443 |
| Pentadecanoate (C15:0) | 3.28±0.33 | 3.28±0.30 | 3.48±0.24 | 0.142 | 0.869 |
| 10-Transpentadecenoate (C15:1T) | 4.14±0.18 | 4.26±0.21 | 4.33±0.16 | 0.268 | 0.769 |
| 10-Pentadecenoate (C15:1) | 68.89±3.57 | 62.90±2.90 | 71.34±2.76 | 1.963 | 0.183 |
| Palmitate (C16:0) | 761.21±98.08 | 700.12±70.15 | 692.73±70.88 | 0.217 | 0.808 |
| Palmitelaidate (C16:1T) | 9.01±0.83 | 8.12±0.60 | 8.91±0.78 | 0.431 | 0.659 |
| Palmitoleate (C16:1) | 233.35±39.05 | 191.98±24.89 | 176.44±19.09 | 1.035 | 0.385 |
| Heptadecanoate (C17:0) | 5.36±0.48 | 5.16±0.40 | 5.73±0.36 | 0.482 | 0.629 |
| 10-Transsheptadecenoate (C17:1T) | 3.86±0.34 | 3.90±0.31 | 3.73±0.26 | 0.08 | 0.924 |
| 10-Heptadecenoate (C17:1) | 18.46±0.68 | 20.52±0.51 | 21.15±1.09 | 3.101 | 0.082 |
| Stearate (C18:0) | 363.02±65.50 | 315.98±42.79 | 395.95±44.16 | 0.6 | 0.564 |
| Elaidate (C18:1N12T) | 6.01±0.53 | 5.89±0.42 | 5.68±0.30 | 0.157 | 0.856 |
| Transvaccenate (C18:1N9T) | 8.61±0.71 | 8.37±0.52 | 7.99±0.42 | 0.305 | 0.742 |
| Petroselinate (C18:1N7T) | 6.35±0.32 | 6.29±0.26 | 6.43±0.34 | 0.052 | 0.949 |
| Oleate (C18:1N9C) | 995.18±106.01 | 958.94±95.21 | 886.83±76.43 | 0.349 | 0.712 |
| Vaccenate (C18:1N7) | 107.16±12.29 | 95.92±7.96 | 95.98±7.55 | 0.463 | 0.64 |
| Linoelaidate (C18:2N6T) | 1.74±0.12 | 1.70±0.08 | 1.57±0.10 | 0.736 | 0.499 |
| 7-Transnonadecenoate (C19:1N12T) | 1.30±0.21 | 1.34±0.16 | 1.24±0.16 | 0.079 | 0.924 |
| Linoleate (C18:2N6) | 459.02±63.53 | 459.60±50.38 | 439.71±43.14 | 0.046 | 0.956 |
| Arachidate (C20:0) | 7.87±0.62 | 7.31±0.49 | 8.32±0.37 | 1.009 | 0.393 |
| *Gamma* Linolenate (C18:3N6) | 4.30±0.50 | 4.93±0.52 | 4.26±0.37 | 0.644 | 0.542 |
| *Trans* 11-Eicosenoate (C20:1T) | 4.46±0.18 | 4.88±0.27 | 4.61±0.18 | 0.966 | 0.408 |
| 11-Eicosenoate (C20:1) | 18.85±2.03 | 17.72±1.49 | 15.56±1.09 | 1.117 | 0.359 |
| Alpha Linolenate (C18:3N3) | 13.80±2.22 | 13.19±1.83 | 11.77±1.28 | 0.33 | 0.725 |
| Heneicosanoate (C21:0) | 1.23±0.04 | 1.17±0.03 | 1.24±0.05 | 0.893 | 0.435 |
| 11- 14 Eicosadienoate (C20:2) | 16.50±0.84 | 15.41±0.96 | 13.28±1.23 | 2.569 | 0.118 |
| Behenate (C22:0) | 21.36±0.87$^{ab}$ | 18.00±0.95$^{b}$ | 21.94±0.94$^{a}$ | 5.325 | 0.022* |
| HomogammaLinolenate (C20:3N6) | 36.41±1.11$^{ab}$ | 40.04±1.48$^{a}$ | 34.69±1.41$^{b}$ | 4.158 | 0.042* |
| Brassidate (C22:1N9T) | 23.50±1.06$^{ab}$ | 21.91±0.46$^{b}$ | 25.50±0.62$^{a}$ | 5.672 | 0.018* |
| Erucate (C22:1N9) | 120.11±7.89$^{ab}$ | 109.39±3.38$^{b}$ | 137.14±7.34$^{a}$ | 4.607 | 0.033* |
| 11- 14- 17 Eicosatrienoate (C20:3N3) | 0.89±0.05 | 0.96±0.04 | 0.91±0.05 | 0.627 | 0.551 |
| Arachidonate (C20:4N6) | 106.26±3.88$^{b}$ | 105.37±4.82$^{b}$ | 140.87±5.64$^{a}$ | 17.537 | <0.001*** |
| Tricosanoate (C23:0) | 0.52±0.02 | 0.51±0.01 | 0.54±0.01 | 1.17 | 0.343 |
| Docosadienoate (C22:2) | 3.34±0.10 | 3.11±0.08 | 3.34±0.07 | 2.564 | 0.118 |
| Eicosapentaenoate (C20:5N3) | 7.93±0.25 | 7.89±0.16 | 7.86±0.30 | 0.019 | 0.982 |
| Lignocerate (C24:0) | 4.08±0.18 | 3.73±0.14 | 4.35±0.22 | 2.979 | 0.089 |
| Nervonoate (C24:1) | 19.68±0.58$^{ab}$ | 18.82±0.81$^{b}$ | 23.15±1.36$^{a}$ | 5.579 | 0.019* |
| Docosatetraenoate (C22:4) | 33.91±1.62$^{b}$ | 32.00±1.75$^{b}$ | 43.18±1.69$^{a}$ | 12.537 | 0.001** |
| Docosapentaenoate (C22:5N6) | 9.69±0.32$^{b}$ | 8.96±0.28$^{b}$ | 13.95±0.81$^{a}$ | 25.799 | <0.001*** |
| Docosapentaenoate (C22:5N3) | 14.73±0.48$^{b}$ | 13.77±0.47$^{b}$ | 18.77±1.04$^{a}$ | 13.726 | <0.001*** |
| Docosahexaenoate (C22:6N3) | 20.51±1.05 | 18.97±0.34 | 19.03±1.46 | 0.68 | 0.525 |

The *F*-value and *p*-value are based on one-way analysis of variance followed by Tukey's multiple comparisons test. The parameters labeled with identical superscript letters (a, b, c) did not exhibit significant differences ($p > 0.05$). *$p < 0.05$; **$p <$

0.01; ***$p < 0.001$. Data are presented as Mean ± Std. Error of Mean (SEM).

**Table S5. Alpha diversity index of gut flora in white feather broilers**

| Items | Treatment | | | *p* |
|---|---|---|---|---|
| | CON(n=5) | L(n=5) | H(n=5) | |
| Goods Coverage | 0.9952±0.0004 | 0.9928±0.0034 | 0.9926±0.0029 | 0.58 |
| Chao1 | 1,092.00±42.68 | 1,254.00±301.00 | 1,316.00±237.60 | 0.58 |
| Shannon | 7.35±0.05 | 7.11±0.11 | 7.33±0.13 | 0.29 |

The *p*-value are based on one-way analysis of variance followed by the Kruskal–Wallis test and Dunn's post-hoc test. The parameters labeled with identical superscript letters (a, b) did not exhibit significant differences ($p > 0.05$). Data are presented as Mean ± Std. Error of Mean (SEM).
